# Evaluating the Feasibility of Frequent Cognitive Assessment Using the Mezurio Smartphone App: Observational and Interview Study in Adults With Elevated Dementia Risk

**DOI:** 10.2196/16142

**Published:** 2020-04-02

**Authors:** Claire Lancaster, Ivan Koychev, Jasmine Blane, Amy Chinner, Leona Wolters, Chris Hinds

**Affiliations:** 1 Nuffield Department of Population Health University of Oxford Oxford United Kingdom; 2 Department of Psychiatry University of Oxford Oxford United Kingdom

**Keywords:** technology assessment, cognition, smartphone, mhealth, mobile phone, Alzheimer disease, early diagnosis, feasibility study, ecological momentary assessment

## Abstract

**Background:**

By enabling frequent, sensitive, and economic remote assessment, smartphones will facilitate the detection of early cognitive decline at scale. Previous studies have sustained participant engagement with remote cognitive assessment over a week; extending this to a period of 1 month clearly provides a greater opportunity for measurement. However, as study durations are increased, the need to understand how participant burden and scientific value might be optimally balanced also increases.

**Objective:**

This study explored the *little but often* approach to assessment employed by the Mezurio app when prompting participants to interact every day for over a month. Specifically, this study aimed to understand whether this extended duration of remote study is feasible, and which factors promote sustained participant engagement over such periods.

**Methods:**

A total of 35 adults (aged 40-59 years) with no diagnosis of cognitive impairment were prompted to interact with the Mezurio smartphone app platform for up to 36 days, completing short, daily episodic memory tasks in addition to optional executive function and language tests. A subset (n=20) of participants completed semistructured interviews focused on their experience of using the app.

**Results:**

Participants complied with 80% of the daily learning tasks scheduled for subsequent tests of episodic memory, with 88% of participants still actively engaged by the final task. A thematic analysis of the participants’ experiences highlighted schedule flexibility, a clear user interface, and performance feedback as important considerations for engagement with remote digital assessment.

**Conclusions:**

Despite the extended study duration, participants demonstrated high compliance with the schedule of daily learning tasks and were extremely positive about their experiences. Long durations of remote digital interaction are therefore definitely feasible but only when careful attention is paid to the design of the users’ experience.

## Introduction

Smartphones will benefit the development of clinical interventions for Alzheimer disease (AD) by enabling detailed cognitive phenotyping at scale [1]. Rich, descriptive, and high-frequency data from every cognitive response, taken from precise on-device sensors, will allow us to move beyond the standard summary metrics of reaction time and accuracy provided by conventional neuropsychological assessment [2], facilitating the detection of subtle performance differences [3]. In comparison with repeat in-clinic tests, remote digital data collection using mobile technologies uniquely enables frequent, longitudinal assessment, with a significantly lower participant burden, lower administration cost, and higher potential for deployment at scale [4,5]. By facilitating a *little but often* approach to assessment, smartphones increase the reliability of cognitive profiling, reducing the impact of within-participant variability, eg, in association with daily stressors, mood, and sleep [6-8]. Together, this maximizes the value of smartphones for the detection of preclinical or prodromal disease, encouraging greater and more targeted recruitment to clinical trials for early AD [3,9]. Frequent measurement allows acute longitudinal change in cognitive function to be accurately assessed [10]; therefore, smartphones can contribute to more exact monitoring of disease progression and therapeutic response [1]. Although digital technology promises clear benefits for dementia research, usability is the greatest challenge for these tools to be widely adopted [1]. This research will consequently investigate participants’ views following the longitudinal use of a smartphone measurement platform.

The feasibility of remote, digital cognitive assessment in older adults is commonly assessed according to study compliance [10-13]. Emphasis has been placed on deploying short, frequent assessments, with adherence to a schedule of self-reported function and active cognitive tasks in the range of 72% to 82% when older adults with no objective cognitive impairment were prompted to interact 5 times a day for a week [14,15]. Further support for the acceptability of high-frequency *microinteractions* (<1 min) is evidenced by 77% compliance in older adults prompted via a smartphone notification to complete four assessments at random intervals across each day for a week [16]. However, to maximize the opportunity of remote, digital phenotyping, it is important to establish whether compliance can be sustained over substantially longer study durations (eg, from several weeks up to several years). Reports of 8.1% participant attrition to a 6-month schedule of remote cognitive tests in individuals at increased familial risk of dementia support the promise of digital tools for long-term assessment [17]; however, further research is needed to understand how to promote sustained participant engagement in digital health research.

Despite the significance of participant compliance for the value of research outcomes, there has been limited evaluation of which factors foster continued engagement with repeat, digital cognitive assessment. A focus group discussion between younger and older adults highlighted participant autonomy in scheduling, positive feedback, and specific instructions on how to approach each cognitive task as important factors for engagement [18]. More broadly, for sustaining long-term engagement (3-17 months) with a Web-based health platform, older adults highlighted personalized reminders from the platform, incorporating the tool into their daily routine, and observed variation or progress within the platform as important [19].

This study tested the feasibility of the Mezurio smartphone app, specifically the utility of this tool for significantly longer-term, high-frequency assessment (36 days) than the 7-day assessment periods explored previously [14-16], along with which factors contribute to successful participant engagement. Mezurio contains a collection of novel cognitive tasks, designed and built by the Mezurio research team to facilitate the detection of preclinical AD. These tasks measure long-term episodic memory, language, and executive function through a range of input modalities including voice, movement, and touch. In addition, the tasks follow a comparable *little but often* approach to remote assessment, as tested previously [14,20], with emphasis placed on providing participants autonomy to schedule tasks according to their daily routine. The developers have worked closely with lay older adults (including adults with mild cognitive impairment) to create a research experience anticipated to be both clear and engaging.

Feasibility was objectively tested according to study compliance and participant attrition across the baseline period of assessment, with the longitudinal follow-up at 6 and 12 months still ongoing. Semistructured interviews, focused on themes of approachability, acceptability, and engagement, were used to evaluate which factors contribute to participants’ willingness to engage in daily digital cognitive assessment for approximately a month. This proof of concept provides the first in-depth evaluation of the feasibility of frequent, remote, and digital cognitive assessment in middle-aged adults, with the high proportion of individuals with a familial risk of AD making this a highly relevant sample for Mezurio’s eventual use case as a tool for detecting preclinical disease [21,22]. Research outcomes will be essential for establishing app utility ahead of the inclusion of Mezurio in larger clinical trials for preclinical AD, as well as improving the use of digital tools in health research more broadly.

## Methods

### Participants

Table 1 reports the demographic characteristics of 35 volunteers (34/35, 97% non-Hispanic whites, aged 40-59 years) recruited via post or an email from the Oxford Health National Health Service Trust site of the larger PREVENT dementia program (n=68) [18]. Although a family history of dementia was not an inclusion criterion for this digital substudy, a high proportion (23/35, 66%) of participants reported a first-degree relative with dementia (15/35, 43% AD). The characteristics of the subset of participants (n=20) who completed a semistructured interview following their period of smartphone assessment are also shown in Table 1, with no significant group differences between participants providing feedback and those not completing the interview—independent groups *t* tests (2-tailed): age (*P*=.85) and years of education (*P=.*68); gender (χ^2^_1_=.84, *P*=.36) and immediate family history of dementia (χ^2^_2_=3.30_,_ *P*=.19).

**Table 1:** Demographic characteristics of participants.

Demographics	Total sample (n=35)	Interview subgroup (n=20)
Age (years), mean (SD)	52.57 (5.10)	52.20 (5.15)
Gender (female), n (%)	26 (74)	16 (80)
Years of education, mean (SD)	15.49 (2.74)	15.30 (3.16)
Family history, n (%)	23 (66)	15 (75)

### The PREVENT Dementia Program

PREVENT dementia [23] is an ongoing prospective study that aims to investigate interactions between risk factors for dementia and traditional biomarkers in midlife. Participants were invited to join PREVENT dementia via a number of routes, including the ConCERT-D and *Join Dementia Research* databases, as well as via the study website and social media. Volunteers were screened at the time of initial consent into the PREVENT dementia program to ensure they met the following criteria: (1) aged 40 to 60 years, (2) no diagnosis of dementia according to the Tenth Revision of the International Statistical Classification of Diseases and Related Health Problems criteria, and (3) no known contradiction to a magnetic resonance imaging scan.

### Informed Consent

This study was ethically approved (University of Oxford Medical Sciences Inter-Divisional Research Ethics Committee: R48717/RE001) and is compliant with the Helsinki Declaration of 1975. Written consent was required upon entry to the study.

### The Mezurio Smartphone App

**Figure 1:**
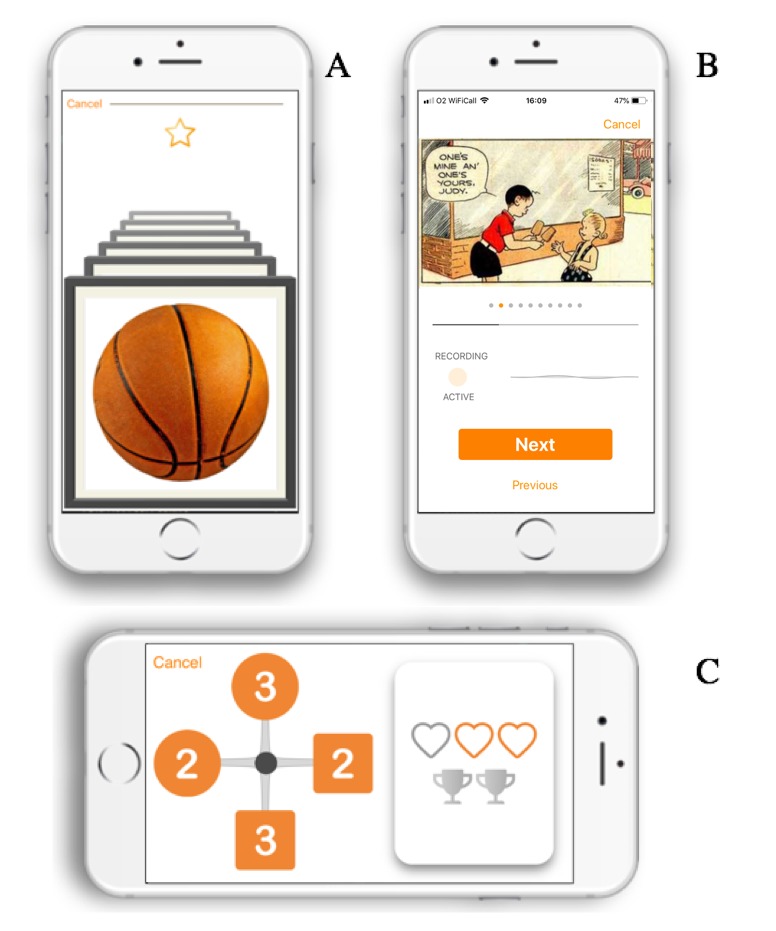
Cognitive tasks deployed within Mezurio: (A) Gallery Game, including the gold star animation presented following a successful learning iteration, (B) Story Time, and (C) Tilt Task, with the participant’s remaining lives (or hearts) and number of levels completed (trophies) shown on the right.

Participants were asked to complete a selection of freely available cognitive tasks within the Mezurio smartphone app, each intended to take 5 min or less to complete. The Mezurio research team designed and built these tasks to measure three core cognitive domains: episodic memory (Gallery Game and Story Time), connected language (Story Time), and executive function (Tilt Task). Figure 1 provides a visual representation of all three tasks. Story Time and Tilt Task were only introduced once a subset of participants (23/35, 66%) had completed their baseline month of assessment, with subsequent recruits (12/35, 34%) asked to complete all three tasks. The first 23 participants were offered the opportunity to switch to this extended version of Mezurio for ongoing follow-up data collection at 6 and 12 months, with 19 participants opting to make this change. No data were collected on why participants did not choose to take part in the extended version of the app.

#### Gallery Game

All participants (n=35) completed Gallery Game during the baseline period of assessment [24], which comprised regular cross-modal paired-associate learning tasks with subsequent tests of recognition and recall memory following ecologically relevant delays (for the current middle-aged population specified as 1, 2, 4, 6, 8, 10, or 13 days). Within each learning activity, participants were asked to encode distinct pairings of object photo–stimuli and touch screen *swipe* directions (left, right, or up), with the number of object-direction associations progressively increasing alongside iterative checks of immediate recall for pairings until the learning criterion was achieved—for more details, see the study by Lancaster et al [24]. Positive feedback (a gold star animation) was presented following each learning iteration if immediate recall was 100% correct (see Figure 1). Incorrect responses were indicated by the object-direction association being cued so the participant could repeat the learning trial, followed by an immediate second test of recall. Participants did not receive explicit feedback on recognition or recall test performance. A number of practice sessions, analogous to the main Gallery Game learning as well as recognition and recall tests, were included at the start of the study schedule. Excluding practice days, participants were prompted to complete up to 22 learning tasks, in addition to the associated recognition and recall tests for encoded stimuli. Participants interacted with Gallery Game once a day for up to 29 days.

#### Story Time

Story Time tests *connected* language; participants were asked to narrate a short comic strip aloud while the device microphone was recording and then repeat the story from memory immediately and following an approximate 24-hour delay. No feedback on performance is provided by Mezurio. Participants were prompted to narrate six comic strips, in addition to the immediate and delayed verbal recall test associated with each. A total of 12 participants completed Story Time during their baseline period of Mezurio assessment.

#### Tilt Task

Tilt Task, a measure of executive function, relies on in-built device movement sensors. Participants were asked to tilt their phone to move the central cursor toward the next target in sequence, with the executive challenge of each presented sequence increasing with successive levels. Inaccurate responses were registered by the central cursor rebounding off the nontarget lure and participants losing one of their three lives. Positive feedback on performance was symbolized by the collection of trophies as participants progressed through the levels. If a participant does not reach the end of the level, the participant cannot move onto the next level.

Note, a number of participants were unable to complete this initial prototype of Tilt Task because of the required sensors (an accelerometer, a gyroscope, and a magnetometer) being absent in a range of Android devices. In addition, a number of participants also reported technical issues with the movement recognition software included in Tilt Task. Consequently, 9 participants were presented with Tilt Task at baseline, with performance data available for only 7 individuals. Subsequent versions of Mezurio screen device sensors when launching the app, with a bug-fix implemented to improve movement recognition during this task.

### Procedure

Participants were invited to download Mezurio onto their personal smartphone (Apple or Android; 28/35, 80%); in cases where the participant’s smartphone was running an incompatible operating system or the participant did not wish to use his or her own device, a number of Android devices were available for loan (n=7). The research team provided written instructions for download and a unique authentication ID.

**Figure 2:**
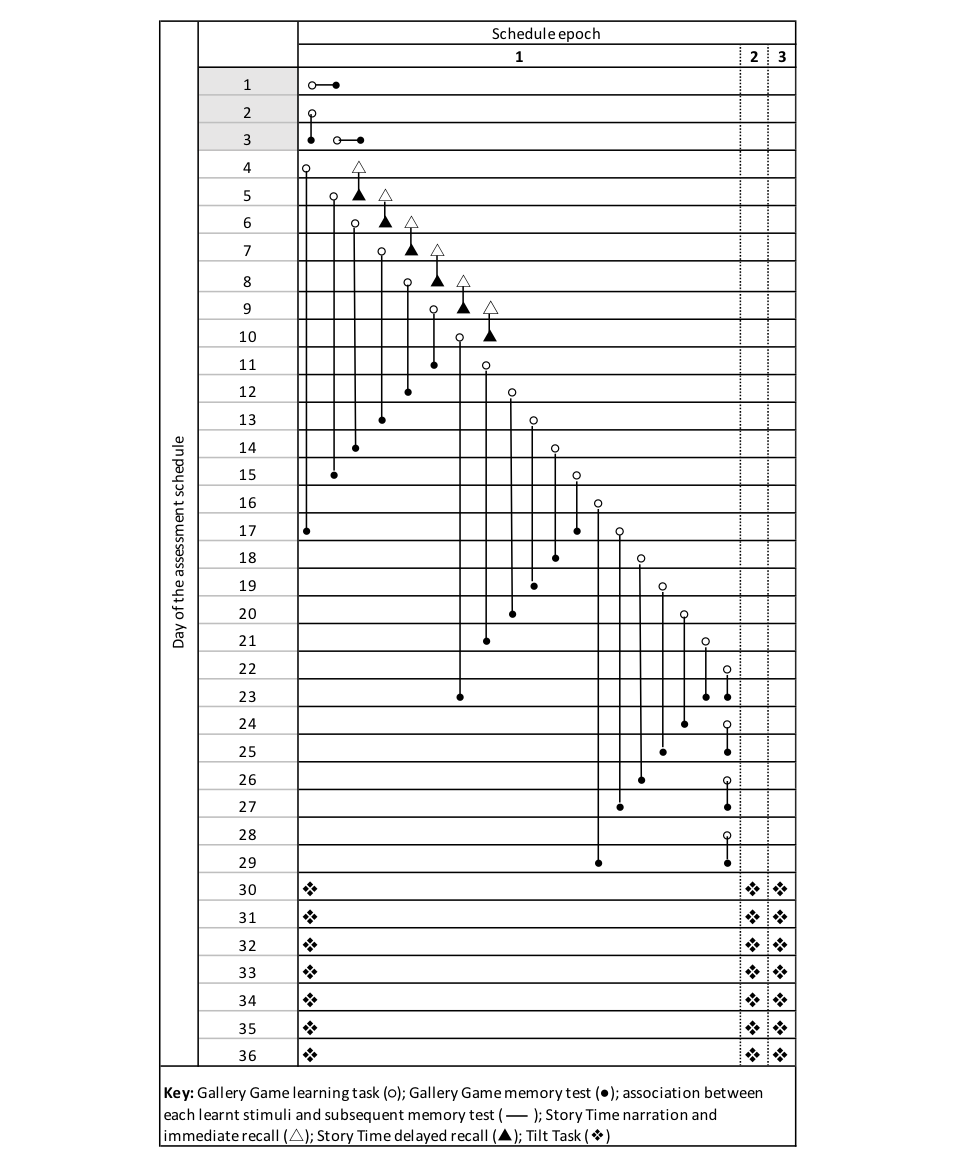
A schematic of the assessment schedule presented to participants completing the extended version of Mezurio, including Gallery Game practice tasks (days 1-3). Schedule epochs represent the three distinct times of the day during which a participant may receive a task notification (eg, after breakfast, lunch, and dinner).

Once installed, Mezurio prompted participants to complete regular cognitive tasks, following a schedule of interactions specified by the app. This schedule is study dependent and can be flexibly tailored according to the research aims and population, with cognitive tasks scheduled for a maximum of 36 days at baseline in this research, dependent on whether participants opted to complete the extended (Gallery Game, Story Time, and Tilt Task) or Gallery Game–only version of Mezurio (see Figure 2). The ongoing follow-up assessment at 6 and 12 months follows a comparable study schedule. The length of study schedule, along with the distribution of tasks within this active test period, was designed to limit daily participant burden while ensuring each individual was presented with multiple opportunities to complete each *microassessment*, anticipated to provide sufficient statistical power for subsequent analysis of cognitive outcomes.

Mezurio typically asked participants to interact once a day, with the exception of individuals opting to complete the extended version of the app, who were additionally prompted to complete Tilt Task thrice daily (see Figure 2). Mezurio encouraged participants to complete the cognitive tasks at the same time each day using local notifications scheduled by the app and displayed on the smartphone’s home screen. A second notification was sent 15 min later if the task was not initiated. Participants chose the time of their scheduled tasks when first opening Mezurio; these notification times could be changed at any time from within the home screen of the app. Following the first task notification, participants had 16 hours to complete each activity before the task *expired*, with the exception of the thrice-daily Tilt Task, which must be completed within 2 hours. Restrictions on task availability were included to both prevent cognitive assessments from accumulating, thereby creating unequal daily participant burden which may adversely impact acceptability, and limit the temporal proximity of task completions (at least eight hours between daily tasks and at least one hour between thrice-daily tasks). The average duration of each task was as follows: Gallery Game learning task (2 min, 16 seconds), Story Time narration (2 min, 17 seconds), and Tilt Task (5 min, 33 seconds).

#### Semistructured Interviews

Participants were invited to provide feedback on their experiences using the Mezurio smartphone app, with the first 20 volunteers selected to complete a semistructured interview (duration: approximately 30 min). Participants who had withdrawn from the study were not invited to complete these semistructured interviews. At the time of interview, all participants had completed their baseline period of assessments, with 13 participants having completed or in the process of completing their 6-month follow-up. As a result of some participants completing the interview after the start of their longitudinal follow-up, only 4 participants had experience using a Gallery Game–only version of Mezurio; however, 3 participants were further unable to interact with Tilt Task as their smartphone did not have adequate sensors (a study smartphone was loaned to participants with an incompatible personal device before the next wave of longitudinal data collection). The schedule of interview questions (Multimedia Appendix 1) included a mixture of closed-answer questions requiring a score out of 10 and open-ended questions, with prompts tailored to each participant’s responses. The presentation of questions broadly mapped to the order in the schedule, with revisions according to individual responses. Interviews were audio recorded and transcribed verbatim.

### Data Analysis

#### Compliance

The proportion of Gallery Game learning tasks attempted by each participant at baseline is considered as the primary outcome of compliance, as this aspect of the study schedule was uniformly presented to all participants. The presentation of Gallery Game memory tests (recognition and recall) was conditional on successful completion of the associated learning task; therefore, compliance with this aspect of Gallery Game was not considered as a separate, independent outcome. The association between participant characteristics and compliance with Gallery Game learning tasks (reverse-score square root transformation to account for negative skew) was screened, using between-group 2-tailed *t* tests for categorical (immediate family history and gender) and simple linear regression for continuous (age and years of education) variables (alpha=.05). Attrition to study participation was proxied as how far through the schedule of Gallery Game learning tasks were participants last active. To further examine whether participation declined as a function of time in the study, the relationship between the assessment index (1-22) and the proportion of participants attempting each scheduled learning task was subject to a nonparametric correlational analysis.

The proportion of Story Time narration (n=12) and Tilt Task (n*=*7) attempted by each participant is also reported; however, the small number of individuals exposed to these assessments at baseline precludes meaningful interpretation. The impact of participant characteristics and time in the study on compliance is not subject to statistical tests because of the limited sample size, but descriptive statistics are provided for the proportion of participants interacting with these tasks each day.

#### Interviews

Interviews were subject to a thematic analysis [25], with the lead author (CL) taking a deductive approach to analyze the transcripts, focused on the following research themes: (1) approachability of the Mezurio app, including a more general consideration of smartphone technology, (2) acceptability of the research ask, and (3) engagement with the cognitive tasks. Additional themes emerging from the data were considered, but these are not considered here as factors contributing to the feasibility of a smartphone-based assessment. The lead author (CL) read each transcript iteratively to extract words and phrases representing the key themes of participant experience.

## Results

### Compliance

**Figure 3:**
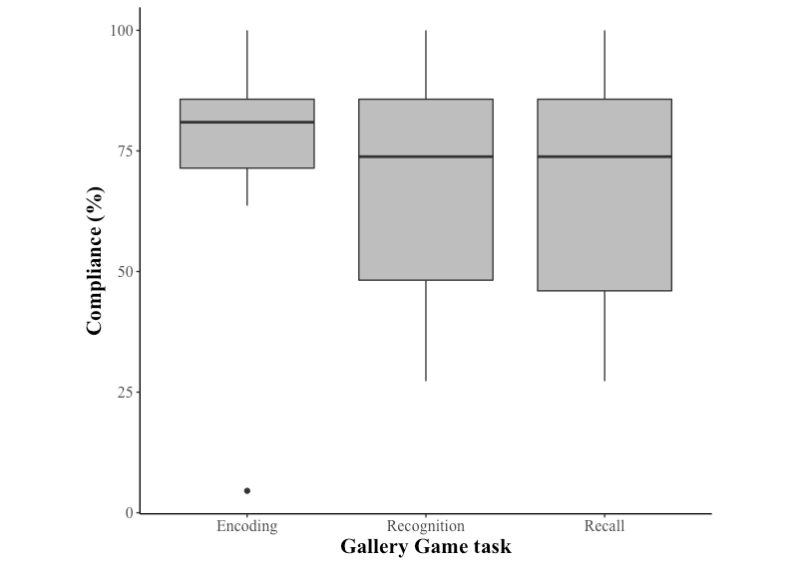
Proportion compliance for Gallery Game tasks (learning, recognition, and recall) across the baseline month of assessments.

Average compliance to the schedule of Gallery Game learning tasks was 77.85% (SD 15.88). One outlier (4.55%) only completed the first learning task, later withdrawing from the study because of the time commitment. Excluding this participant, average compliance was 80.00% (SD 9.60). Volunteer characteristics are shown in Table 1. An immediate family history of dementia (mean 79.13, SD 9.33) was associated with poorer compliance to the study schedule in comparison with those with no family history (mean 81.79, SD 10.38;tt_43.33_=17.25; *P*<.001), as was being female (mean 79.30, SD 9.38) as opposed to being male (mean 82.30, SD 10.62;t_41.67_=12.41; *P*<.001). However, these associations must be considered with caution because of limited group sizes. There was no significant association between age (*P*=.10) and years of education (*P*=.74) with compliance to the schedule of Gallery Game learning tasks. Participants completed an average of 67.26% (SD 20.61) of recognition tests and 66.63% (SD 20.59) of recall tests (see Figure 3).

Attrition across the baseline period of Gallery Game learning tasks was limited, with 88% of the participants completing the final scheduled learning task. The mean proportion of the schedule completed at the point of final learning was as follows: 98.15% (SD 5.70); range 75% to 100%. In addition, the percentage of participants compliant with each of the 22 scheduled learning tasks presented across the baseline assessment period is shown in Table 2. There was a nonsignificant, limited relationship between the learning task index and compliance (=0.09; *P*=.60).

Average compliance to the schedule of story narration tasks was 75.00% (SD 26.11), with the proportion of participants attempting each of the six scheduled daily tasks as follows: (1) 83%, (2) 92%, (3) 75%, (4) 75%, (5) 67%, and (6) 58%. Participants completed 38% (SD 31.83) of thrice-daily Tilt Tasks; however, the range of tasks (1-17) attempted by participants (out of a possible 21) may reflect the technical problems some participants reported for this task. The proportion of participants active each day of Tilt Task was as follows: (1) 100%, (2) 71%, (3) 57%, (4) 43%, (5) 29%, (6) 57%, and (7) 29%, but note the very small sample size (n=7).

**Table 2:** The proportion of participants (n=35) completing each of the 22 Gallery Game learning tasks distributed across the baseline Mezurio assessment period.

Task index	Compliance (%)
1	97
2	89
3	91
4	94
5	86
6	80
7	83
8	83
9	83
10	89
11	89
12	89
13	94
14	89
15	80
16	86
17	83
18	77
19	67
20	83
21	67
22	83

### A Thematic Analysis of App Experience

Quotations evidencing the extracted themes are present in Multimedia Appendix 2.

#### Approachability of the Mezurio App

##### The Approachability of Smartphones

The approachability of smartphones was confirmed in this study’s middle-aged sample, with all interviewed participants owning a personal device (12 Apple iPhone users and 8 Android users) and 90% of the participants reporting daily or more frequent smartphone usage (the remaining participants stated they used their phone on most days). Indeed, 35% (7/20) of participants reported active attempts to decrease smartphone usage in their daily lives, supporting the prevalence of these technologies in the target population, with one participant stating:

It’s a double thing where by the convenience is amazing but it's also very intrusive, and I have to consciously switch it off or put it away or put the ringer off or something for it to not be constant.

OX017

Comparable smartphone ownership is inferred in the wider study population (n=35); the cited reasons for borrowing a study device (n=7) were not wishing to use their personal phone for the purpose of the study (n=2) and smartphone incompatibility because of either an outdated operating system (n=2) or inadequate sensors for the completion of Tilt Task (n=3), rather than participants not owning a device. Smartphones were used by participants for a wide range of functions, including communication, work, finance, travel, and shopping; however, 35% (7/20) of the participants reported viewing their phone as a functional tool rather than a device used for enjoyment. The majority (15/20, 75%) of this middle-aged population did not use phones for gaming, with the two reasons reported for this being time commitment (3/20, 15%) and lack of interest (3/20, 15%). A small number of participants had previously completed the AD-focused SeaHero Quest (2/20, 10%) [26] or used *brain-training* apps (2/20, 10%).

##### Remote Setup

Remote setup of the Mezurio app was well accepted, with the clarity of the installation process scoring an average of 9.03 out of 10 (SD 1.52); participant OX066 recalled the Mezurio setup process as:

I clicked the click and in....

A total of 80% (16/20) of the participants reported that the written instructions for installation were sufficient; indeed, 1 participant felt the quantity of written guidance provided by the research team was a barrier to completing what in reality was a very simple task, stating the following:

it was sufficiently information intensive to make me feel like I had to put it down and find time to do it whereas successful calls to action just make it super easy for you to go click, click, done... I think, I think more information is not necessarily better communication.

OX037

##### Task Onboarding

Task onboarding for Gallery Game was appropriate for this sample, with instructions scoring an average of 9.30 out of 10 (SD .10). Participants highlighted a number of features that were effective for remote introduction to Gallery Game, including simple, short written instructions, the inclusion of *pop-up* responsive help within the task, and the provision of a task strategy at the start of the assessment period. Participants were allowed up to 7 days to practice the Gallery Game task; the consensus (13/20, 65%) was that an initial practice period of 1 or 2 days is sufficient for understanding task demands. For participants completing Tilt Task, the instructions were again judged to be clear, with participants highlighting being provided with an opportunity to practice, coupled with a gradual increase in task complexity, as important for task onboarding; OX011 stated the following:

and the way it gradually increases with complexity. I mean, that's a hard thing to describe because it's one of those things you just really need to see and try it and then the instructions kind of make sense if you see what I mean.

There was less consensus around the clarity of task onboarding for Story Time; participants were unclear of the amount of descriptive detail to provide during narration:

they say describe in as much detail as possible erm, but I'm not sure you really do want that because somebody who takes things so literally, like me, and then I spend forever doing the first scene.

OX066

##### The User Interface

The user interface of Gallery Game and Tilt Task was acceptable, with the manual response mechanism of the Tilt Task evaluated positively by 40% (8/20) of participants. Qualitative feedback suggested that the spoken interface of Story Time increased the burden of remote, digital assessment (7/20, 35%) by limiting situations in which the task could be completed, with participant OX015 explaining:

it was just another layer of something I had to do to complete it, you know, I had to take myself of to a room or a quiet space to do it...

Across Mezurio, visual clarity of the app was highlighted (6/20, 30%) as an important feature, with OX026 stating:

I liked the layout because it’s clear, it’s not, sort of, forced into a scrumpled little heap that you have to read carefully. It's well laid out and the writing is a good size to read...

#### Acceptability of the Research Ask

The approach in this study to remote cognitive assessment was scored 8.37 out of 10 (SD 1.34) for participant acceptance. The limited number of participants (4/20, 20%) completing the Gallery Game–only version of Mezurio precludes traditional significance testing; however, the mean acceptability of this group (mean 9.00) is comparable with ratings by those completing the extended version of the app (mean 8.20).

##### Daily Time Commitment

Daily time commitment was an important feature in the acceptability of the current participation ask, with short task durations being important in allowing the app to work around participants’ schedules:

I think having it short is good because if you make it too long then you're going to find that people like me, if we're out at work then trying to fit in makes it more of a problem.

OX062

Increased daily participation load in the extended version of Mezurio, characterized by occasional, additive scheduled activities (eg, a Gallery Game and Story Time on the same day) and longer daily task durations contributed to an increased sense of burden (3/20, 15%). This study asked participants to interact with the app every day for up to 36 days; this study duration was not a significant problem for the current research group, with limited (3/20, 15%) discussion of this as an issue.

##### Scheduling the Tasks

Scheduling the tasks via local phone-based notifications benefitted study compliance (n=20), with many participants noting that the regularity of app timings helped them complete their daily tasks, eg, OX033 stated:

the fact that it was very regular, it was once a day, such a regular thing, it just became part of my daily, you know, habit.

Specifically, the inclusion of short, cognitive assessments within the participants’ daily routine was highlighted as important, with poor compliance generally accounted for by changes to an individual’s regular patterns of activity, eg, weekends and trips (3/30, 15%). Participants using the extended version of Mezurio were prompted to complete Tilt Task thrice daily, with the alignment of task notifications to the daily routine (eg, breakfast, lunch, and dinner) reported to be increasingly important for high-frequency assessment. A total of 3 participants reported finding the 3-times-a-day routine to be too much to fit into their daily routine.

##### Flexibility of the Study Schedule

Flexibility of the study schedule was emphasized as a requisite for high-frequency, remote assessment. Within the app, participants had the option of changing the time of their next prompt; 7 participants identified this feature as a strength, with participant OX066 stating:

...really nice that you could change the times. I got quite good at thinking that's not going to work tomorrow and changing them.

Participants (5/20, 25%) suggested that Mezurio could be improved in the future through the inclusion of a *snooze* function for notifications, allowing participants to set a second reminder for later in the day. Actively maintaining the intention to complete Mezurio after noncompliance with the initial prompt was associated with increased subjective burden (2/20, 10%). Presently, participants are able to re-enter the Mezurio app and complete their daily task within a certain expiry window (once-daily tasks: 16 hours; thrice-daily tasks: 2 hours). This feature benefitted research acceptability; however, there was still a wish for greater flexibility. A further suggestion to improve Mezurio is the ability for participants to monitor the task expiry time (3/20, 15%).

##### Perceived Burden

Perceived burden of the research ask was largely dependent on the external stressors to a participant’s time, independent from the app, which is explained as follows:

I mean, conceptually it wasn't burdensome it was absolutely fine, you know, I didn't feel I was being put upon, erm, practically, however, I just found that the, my ability to, err, err, to, to, to adhere to the schedule... just got compromised by the ins and outs of daily life.

OX037

Furthermore, daily-life stressors were associated with subjective reports of poorer cognitive performance on that day, supporting the need to sample cognition at multiple time points to maximize reliability. Asking participants to interact with the app thrice daily was considered burdensome by some participants (3/20, 15%).

#### Engagement

##### Task Enjoyment

Task enjoyment was scored out of 10 as following: Gallery Game mean 7.26 (SD 2.02), n=19; Tilt Task mean 6.44 (SD 2.62), n=12; and Story Time mean 7.18 (SD 1.49), n=14. The cognitive demand of included assessments was cited as a primary source of participant enjoyment (9/20, 45%), with participants reflecting on the satisfaction of challenging themselves in comparison with their previous performance and the development of personal strategies to aid performance (3/20, 15%):

I was surprised how...a very simple task, task, was actually quite hard to do and so I could feel it challenging my brain, and that, that felt good.

OX066

In contrast, barriers to research enjoyment included concern over their own performance (6/20, 30%), frustration at difficult tasks (4/20, 20%), and limited variation in the day-to-day task demand (2/20, 10%):

by the last one I just felt “how many more!”...I guess there will be reasons why you've done several days in a row but if you could do sev, a few days and then a break, and then come back and do a few days.

OX042

Enjoyment was not considered an important factor for compliance by a small number of participants describing the tasks as functional as opposed to fun. In relation to this, a main motivator for engagement was commitment to the research aims rather than personal pleasure.

##### The Inclusion of Feedback

The inclusion of feedback was identified by participants as the foremost way to promote participant engagement with remote cognitive assessment. Although limited explicit feedback on performance outcomes is provided in the current version of Mezurio, participants had an intuitive sense of their own performance (7/20, 35%). Participants highlighted a need for explicit feedback on how their performance changed across the course of the research, with feedback on performance in comparison with peers identified as a potential factor to increase study participation. In addition, greater dissemination of the research background, objective, and methods within the app was highlighted as a potential to promote further engagement:

...it would be really helpful to get some commentary on what it's contributing to, even if that needs to be left until the end...but within the game. It's not to get an email, you know a week later with a 2-page pdf, that's mainly because I just won't read it....

OX037

## Discussion

### Principal Findings

This study tested the feasibility of digital cognitive assessment, deployed through the Mezurio smartphone app, in a middle-aged group relevant to the detection of early preclinical AD [22,23]. Participants were prompted to complete a prolonged schedule of daily assessments (36 days); a qualitative evaluation of Mezurio’s user interface and task design, alongside study compliance and attrition, was used to explore whether smartphone-centered tools can substantially extend the breadth of cognitive assessment in which participants will engage. Excluding 1 participant who subsequently withdrew because of the time commitment, compliance with the schedule of Gallery Game learning tasks averaged 80%, with 88% of the participants still active at the end of the assessment period (36 days), confirming the feasibility of frequent, long-term cognitive assessment in a digital environment. Critically, participant feedback supported the acceptability of Mezurio’s approach to digital assessment, with an intuitive user interface, flexible scheduling around personalized prompts, and engagement within the tasks themselves identified as important factors contributing to a positive research experience. Preliminary reports of compliance with the schedule of story narration and tilt executive function measures were at 75% and 38%, respectively, but the small number of individuals exposed to these activities at baseline and the technical issues identified in this first deployment of Tilt Task limit interpretation of these data.

Importantly, this study demonstrated participant engagement with daily cognitive assessments across a significantly longer study duration than the previously reported 7-day window [14-16]. This provides evidence that smartphone technologies enable far greater sampling of cognition than plausible with in-person tests. Consistent with the high levels of participation reported in older adults who are asked to complete frequent mobile cognitive assessments [14-16], high compliance in this middle-aged group confirmed the feasibility of a *little but often* approach to smartphone-based cognitive testing. Furthermore, limited attrition supports the potential utility of smartphone-based tools for long-term monitoring. Participation *dropout* is reported in digital research spanning multiple months [17,27], with cited reasons for withdrawing from the research including technical problems, time commitment, and loss of interest in repetitive tasks [27]. Although study reuptake at months 6 and 12 for this initial pilot of Mezurio remains to be established, the progression of strategies to sustain participant engagement is important for the quality of collected data. In addition, the generalizability of research outcomes must be considered, with the limited published data in this field suggesting compliance to a schedule of remote cognitive assessment differs between study groups; 27% compliance has been reported in young adults [28] compared with 84% to 91% compliance in adults with reported substance abuse [29,30].

A major benefit of smartphone assessment is the ability for participants to independently contribute to research from a home environment; therefore, it is critical to establish the remote usability of such measures before implementing them at scale. Middle-aged participants in this study positively evaluated the clarity of setup and task instructions for Mezurio, with concise written instructions, an opportunity to practice, and responsive inbuilt help identified as design strengths—developed through iterative patient and public involvement. All participants were frequent smartphone users; however, although the adoption of smartphone technology is increasing in older age groups (49% of adults in the United Kingdom aged 55-64 years and 17% of adults aged over 65 years reported owning a device in 2015) [31], generalizability of app usability remains to be established. Subjective feedback from participants, including those with no previous experience with smartphones, on a smartphone app for monitoring the symptoms of chronic pain identified inexperience with technology and the need for technical support as potential issues [32]. Future work will further establish the feasibility of Mezurio in a wider general population.

Scheduling, both the length of individual assessments and participant autonomy in the timing of smartphone-based notifications, was critical for the acceptability of reseach ask [10,18,19]. Each task within Mezurio is designed to take 5 min or less to complete; although acceptability of the current participation ask was high (8.37/10), time commitment was reported as a primary reason for noncompliance, emphasizing the value of a *little but often* approach for repeat mobile assessment [14-16]. The allowance to personalize phone-based notifications to suit participants’ daily routine and the flexibility given to delay responding to scheduled assessments were identified as important factors in limiting research burden by participants providing discursive feedback. Although the current version of Mezurio does not objectively record the scheduling behavior of participants, including notification times and the lag between participants receiving such a prompt and initating their next task, this may be explored in future research to improve the utility of Mezurio for time-sensitive research protocols, eg, monitoring treatment response.

The mental challenge of daily activities was identified as an important factor for participant engagement with repeat Mezurio assessments, with the suite of tasks within this app intended for use by adults with no clinical diagnosis of cognitive impairment in contrast to a more traditional approach to neuropsychological assessment. However, in a limited number of participants, subjective performance was associated with anxiety, which is relevant for the future modification of these tasks for use in individuals with mild cognitive impairment. Presently, Mezurio provides limited explicit feedback on task performance, with participants suggesting that future personalization of this aspect of the app would benefit sustained participant engagement, consistent with previous focus groups [18,19]. In accordance with Mezurio’s intended screening utility for preclinical dementia, careful consideration of how to ethically communicate performance outcomes to participants is needed. Importantly, feedback in this study suggested that short communications within the app, intended to inform and promote interest in the research area, may similarly contribute to long-term engagement.

### Limitations

A limitation of this study is that the Tilt Task and Story Time were included as an optional, later amendment, meaning that participants did not have a uniform experience of using the app. In addition, the reasons were not evaluated for participants declining to take part in this extended version of Mezurio at 6-month follow-up and for declining or withdrawing from (n=1) this ancillary technology substudy of PREVENT more broadly; this would have provided additional insight into the approachability of smartphone cognitive assessment. In addition, a number of participants experienced technical issues, which prevented or disrupted their completion of Tilt Task assessments, likely to impact reported Tilt Task compliance and subjective feedback. Building on this early, pilot study, a number of improvements have been made to the software underpinning this task.

When drawing on these results, it is worth noting that this study’s sample was small and recruited from an existing, more intensive prospective cohort. Specifically, as these participants were *research aware* and had already demonstrated a high commitment to dementia research, mirrored in their qualitative feedback, the promise of using digital biomarkers to screen cognition remotely may not generalize to a wider population. The GameChanger study [33] has been launched to directly investigate whether the feasibility of Mezurio generalizes across a wide UK demographic, with over 16,000 participants completing remote high-frequency cognitive assessments within the app to date. It is also notable that the recruited sample comprised cognitively healthy individuals; thus, the acceptability and feasibility of using the app in impaired populations remains to be studied. Conclusions from this study, along with further input from patient and public involvement sessions, have been used to strengthen the research design implemented in the Mezurio smartphone app.

### Conclusions

This research supports the feasibility of the Mezurio smartphone app for extensive cognitive profiling in middle-aged adults, evidencing high compliance to a substantially longer schedule of daily interactions than previously explored. The scheduling of smartphone interactions, clarity of user experience, and task design were critical for reported engagement with Mezurio, with the qualitative feedback presented here providing an important direction for implementing digital tools in future health research. Following this initial demonstration of the viability of remote Mezurio assessment as a complementary method to in-clinic assessment, ongoing work seeks to replicate this feasibility in a wider population, as well as in adults with a diagnosis of dementia. In addition, establishing the scientific utility of these novel cognitive tasks in comparison with traditional markers of preclinical dementia (eg, brain-based biomarkers, neuropsychological outcomes, and prospective decline) is still in progress. However, the conclusions in this study are an important first step in justifying a participant-orientated mobile design for the progression of efficient, early screening of cognitive decline.

## Appendix

Multimedia Appendix 1 The schedule for the semistructured interviews.

Multimedia Appendix 2 Thematic outcomes of self-reported research experience with the Mezurio app, evidenced by participant quotations.

## Abbreviations

AD: Alzheimer disease
