# Supplementary material for: Evaluating the Feasibility of Frequent Cognitive Assessment Using the Mezurio Smartphone App: Observational and Interview Study in Adults With Elevated Dementia Risk
Source: JMIR Mhealth Uhealth. 2020 Apr 2;8(4):e16142. doi: 10.2196/16142 (PMC7163418; doi:10.2196/16142)
Supplement: Multimedia Appendix 1 [file mhealth_v8i4e16142_app1.docx]

**Multimedia Appendix 1**

1. Was this your first time using a smartphone?
   1. If yes:
      1. Did you enjoy using the smartphone?
      2. Could you describe any problems you had using the smartphone?
   2. If no:
      - If you own a smartphone, is it Android or Apple?
      - How regularly do you use a smartphone?
      - What sort of things do you use your smartphone for?
      - Do you have experience playing games on a smartphone?
      - If the participant says they play games – what sort of games, how often and why? If not, why not?
      - How enjoyable do you find using your smartphone?

**Downloading, installing and setting up the app:**

1. How do you rate the app download process out of 10, with 10 being very straightforward and 1 being very complicated?
   - Why? e.g. was there sufficient information from the research team, any problems downloading and installing the app.

**Instructions and daily notifications:**

1. How clear did you find the instructions for completing the Gallery Game task on a scale of 1-10, with 10 being very clear and 1 being not clear at all?
   - Why? e.g. what was unclear, did you have any problems understanding the instructions?
2. The app sent you a notification each day to remind you to play the game, were these helpful in completing your task?
   - Is there anything else we could do to help you engage with the app every day?

**Gallery Game**

1. The app asked you to complete a task, for a few minutes, every day for about a month. How acceptable or burdensome was this on a scale of 1-10, where 10 is very acceptable, and 1 is very burdensome?
   - Why?
2. Were there any occasions when you weren’t able to complete the task?
   - Why?
3. How enjoyable did you find doing the memory task on a scale of 1-10, with 10 being very enjoyable and 1 being not at all enjoyable?
   - Why?
4. How many practice days did you need before you felt confident completing the task?
5. Did you use a strategy to help you remember photos during the memory task?
   1. If so, can you describe it?
6. How easy or difficult did you find the completing the memory task on a scale of 1-10, with 10 being very easy and 1 being very difficult?
   - Why? e.g. which aspects did you find difficult?

**For people who consented to complete the extended version of Mezurio**

**The Tilt Task**

1. How enjoyable did you find doing the Tilt task on a scale of 1-10, with 10 being very enjoyable and 1 being not at all enjoyable?
   - Why?
2. Did you feel the practice days were sufficient for you to understand the task?
3. How easy or difficult did you find the completing the Tilt task on a scale of 1-10, with 10 being very easy and 1 being very difficult?

- Why? e.g. which aspects did you find difficult?
- Aspects of the task you would improve if they say it was difficult for technical/app-based reasons rather than the cognitive demand of the task itself?

**Story time**

1. How enjoyable did you find doing the Story time on a scale of 1-10, with 10 being very enjoyable and 1 being not at all enjoyable?
   - Why?

Suggestions for improvement if found the task not so enjoyable

1. How easy or difficult did you find the completing the memory part of Story time on a scale of 1-10, with 10 being very easy and 1 being very difficult?
   - Why? e.g. which aspects did you find difficult?

**General Questions:**

1. How would you rate the experience of using the app out of 10, with 10 being a very positive experience and 1 being a very negative experience?
   - Why?
2. Can you describe any problems or issues you had with the app?
3. What aspects of the app did you like?/dislike?
4. Is there anything we could do to improve your experience of using the app? (Including downloading the app, instructions and the design of the tasks)
