# Supplementary material for: Evaluating the Feasibility of Frequent Cognitive Assessment Using the Mezurio Smartphone App: Observational and Interview Study in Adults With Elevated Dementia Risk
Source: JMIR Mhealth Uhealth. 2020 Apr 2;8(4):e16142. doi: 10.2196/16142 (PMC7163418; doi:10.2196/16142)
Supplement: Multimedia Appendix 2 [file mhealth_v8i4e16142_app2.docx]

**Multimedia Appendix 2**

| **Superordinate themes** | **Themes** | **Quotations** |
| --- | --- | --- |
| Approachability of Mezurio | Smartphone technology | - “It's kind of attached to me” (OX015) |
|  |  | - “every 6 seconds” (OX037 – reporting how often they use their smartphone) |
|  |  | - “It's a double thing where by the convenience is amazing but it's also very intrusive, and I have to consciously switch it off or put it away or put the ringer off or something for it to not be constant” (OX017). |
|  | Remote set-up | - “My memory is just I clicked the click and in. I've downloaded other things as well before and it was just as easy as the easiest of them, I just clicked the click and there it was, it was there.” (OX066) |
|  |  | - “I mean the process was quite straightforward in the sense that there's not much to it, I just had to download it and put the code, that's all, so there's not much to it.” (OX035) |
|  |  | - “it was sufficiently information intensive to make me feel like I had to put it down and find time to do it whereas successful calls to action just make it super easy for you to go click, click, done...I think, I think more information is not necessarily better communication.” (OX037 – on the written instructions provided by the research team) |
|  | Task onboarding | - "I didn't have a problem with it because, because it's, it's a relatively simple task, it is not, there are not a lot of complicated instructions. You look at a picture and you follow an arrow so it's nice and simple in itself.” (OX026-on Gallery Game) |
|  |  | - “I quite liked the, when you were learning, you know when you had, when you went to do it and it was wrong, the reminder, you know, while you were learning, that was really good feedback.” (OX036- on Gallery Game) |
|  |  | - “And the way it gradually increases with complexity. I mean, that's a hard thing to describe because it's one of those things you just really need to see and try it and then the instructions kind of make sense if you see what I mean?” (OX011- on Tilt Task) |
|  |  | - “it isn't exactly, they say describe in as much detail as possible erm, but I'm not sure you really do want that because somebody who takes things so literally, like me, and then I spend forever doing the first scene.... and then I'm thinking later 'I'm sure, that actually, they probably didn't want that much detail" (OX066- on Story Time) |
|  | User-interface | - “I liked the layout because it's clear, it's not, sort of, forced into a scrumpled little heap that you have to read carefully. It's well laid out and the writing is a good size to read… I think this needs to be clear and the bullet points were nice and short so you're not reading too much text at any one time, so again if you, I mean if you're beginning to sort of have, shall we say, problems with, not just memory but erm, time. You know, you have a short span of attention, then, short enough.” (OX026) |
|  |  | - "I think maybe it was just because it felt like it was more of a physical, I know it's not, I know it's just like your hands and things doing a bit, it just felt a bit more active and different to the stuff we'd been doing so, I liked the variety of it I think.” (OX039 – on Tilt Task) |
|  |  | - “It wasn't difficult, it was just another layer of something I had to do to complete it, you know, I had to take myself of to a room or a quiet space to do it, which I didn't have to for the others.” (OX015 – on Story Time) |
| Acceptability of research ask | Time commitment of daily tasks | - “I think having it short is good because if you make it too long then you're going to find that people like me, if we're out at work then trying to fit in makes it more of a problem.” (OX062) |
|  |  | - “And it didn't take too long either. I didn't find the time cumbersome at all, it was just. Because I work full time it was just finding a slot to get it into if you see what I mean.” (OX015) |
|  |  | - “The Gallery Game was fine because generally speaking that took less time than it estimated it would take, erm, but I know some of the later, more complex games, erm, I found it a bit of a struggle because they, they took quite a bit longer and just trying to find time in the morning is, err, not always easy.” (OX011) |
|  | Scheduling the tasks | - “I think because I had it sort of first thing in the morning, it was something I'd look forward to, sort of getting up and doing, but also having a little reminder, that was good.” (OX039) |
|  |  | - “the fact that it was very regular, it was once a day, such a regular thing, it just became part of my daily, you know, habit.” (OX033) |
|  |  | - "3 times a day was about right. For all the tasks really, coz you used to like set it up for morning before work, when you're at lunch and when you get back home again so I found that quite, quite easy. It was a good way of trying to sort of, you know get the most out of the subject, i.e. me, but without putting too much pressure on them to do it.” (OX062) |
|  |  | - If it's a normal day and it falls in your routine that's fine. And then as soon as you have to go and do something differently on a day it becomes a bit more of a nuisance…So therefore, you possibly need to have that flexibility as to when you're going to do it.” (OX023) |
|  | Schedule flexibility | - “Very, very, and really nice that you could change the times. I got quite good at thinking that's not going to work tomorrow and changing them.” (OX066) |
|  |  | - “some kind of snooze, that would be, erm, that would be very helpful, or, I didn't complete it as often as I would of wanted to and it was almost certainly every time because I was in the middle of something and by the time I'd got out of doing what I was doing, I, you know, it had just slipped my mind.” (OX037) |
|  |  | - “There were a couple of times when I did change the time of day when I was playing it, and that might of been because I needed to do a piece of work, or I needed to be somewhere else at that time of day, particularly at weekends. When, you know, you might be doing something else.  Erm, so it was useful for that time not to be absolutely rigid.” (OX013) |
|  |  | - “'yes I should be free for say 9 o'clock tomorrow' and then something happens and you overrun and it gets to 10 o'clock and there are a couple of times when it, it sort of, I think sulked and if I was late it just didn't start.” (OX062) |
|  | Perceived burden | - “I mean, conceptually it wasn't burdensome it was absolutely fine, you know, i didn't feel I was being put upon, erm, practically, however, I just found that the, my ability to, err, err, to, to, to adhere to the schedule... just got compromised by the ins and outs of daily life.” (OX037) |
|  |  | - “I found my memory was affected by things, err, so if I'd had a late night, erm,... my memory definitely wasn't, erm, as good, erm, or if something stressful had been going on, you know, that sort of, it sort of put me of a little or I just didn't remember things as well.” (OX033) |
|  |  | - “when it gets to three times a day, it does come, like, quite a commitment. To be able to fulfil it. I'm not saying it's impossible but it's just a big commitment.” (OX023) |
| Engagement | Task enjoyment | - “I was surprised how... a very simple task, task, was actually quite hard to do and so I could feel it challenging my brain, and that, that felt good.” (OX066) |
|  |  | - “I'm quite interested in how the memory works and so on. And so, having to device my own strategy that I then found made the task a lot easier, that added to an enjoyment I suppose.” (OX011) |
|  |  | - “by the last one I just felt 'how many more!' it just felt like I was doing an awful lot of storytelling into my phone… I guess there will be reasons why you've done several days in a row but if you could do sev, a few days and then a break, and then come back and do a few days.” (OX042) |
|  |  | - “It's a complete random guess and then you just end up feeling a bit stupid because you think well I'm just doing a random, I can't even remember seeing this item so how can I remember what direction it goes in.” (OX026) |
|  |  | - “I feel if it's helping you and it's helping the research then it makes me feel good. I felt it was er, an easy way of providing what I hope is useful information.” (OX038) |
|  |  | - “I think that if, erm, it wasn't something I had committed to, you know, for a number of reasons, I think if it was just on my own benefit, just for fun, I probably wouldn't have, erm, you know carried it on, so there was that sort of, compulsion, I suppose.” (OX033) |
|  | The inclusion of feedback | - “obviously I had some feedback here that I was getting better, but I, in the sense that you got to the number 24 eventually rather than starting it, finishing it at number 8. So, yeh I'd like to play that longer because I just saw it as a challenge that I wasn't very good at.” (OX038 - on Tilt Task) |
|  |  | - “I suppose also, if you sort of, perhaps with the results, if there was some way of you sort of like archiving it so you could see your results over a period of time… so you could see the trend, if you're doing better or worse. But then that depends obviously on the people, because, because I'm sort of a bit competitive to me that would help improve and for other people, it may cause some issues if they see that they're not improving.” (OX062) |
|  |  | - "it's kind of erm, dates in a vacuum, it would be really helpful to get some commentary on what it's contributing to, even if that needs to be left until the end…. but within the game. it's not to get an email, you know a week later with a 2-page pdf, that's mainly because I just won't read it… it's in the moment… you've got my attention, you've worked very hard to get my attention….so now you've got my attention in that window of 3 seconds as I'm winding down from the game, just tell me something.” (OX037) |
|  |  | - “I'd love to know more about how you assess the information and what that means.” (OX026) |
